# Polymorphic transition and nucleation pathway of barium dititanate (BaTi_2_O_5_) during crystallization from undercooled liquid

**DOI:** 10.1038/s41598-019-43357-6

**Published:** 2019-05-10

**Authors:** Xuan Ge, Qiaodan Hu, Wenquan Lu, Sheng Cao, Liang Yang, Mingqin Xu, Mingxu Xia, Jianguo Li

**Affiliations:** 1 Shanghai Key Laboratory of Materials Laser Processing and Modification, School of Materials Science and Engineering, Shanghai Jiao Tong University, 200240 Shanghai, P.R. China; 2 Monash Centre for Additive Manufacturing (MCAM), Monash University, Clayton, VIC 3800 Australia

**Keywords:** Materials science, Structural materials

## Abstract

The nucleation pathway plays an important role in vitrification, preparation of glass-ceramic composites and synthesis of metastable materials. In this paper, we studied the nucleation pathway of a novel ferroelectric BaTi_2_O_5_ (BT2) during crystallization from undercooled liquid by aerodynamic levitation (ADL) containerless processing and structural analysis. An interesting polymorphic transition of BT2 regulated by the undercooling was observed during the crystallization process: the ferroelectric monoclinic phase (**γ**-BT2) was fabricated at low undercoolings and the paraelectric orthorhombic metastable phase (**β**-BT2) was obtained from hypercooled liquid. This polymorphic transition phenomenon corresponds to a non-classical nucleation pathway: metastable **β**-BT2 preferentially nucleates from undercooled melt and **γ**-BT2 is generated from **β** phase by solid-state phase transition. The two-step nucleation pathway stems from the structural heredity between the undercooled liquid and crystals. A stronger structural homology exists between the undercooled melt and **β**-BT2 than **γ**-BT2 based on diffraction data and atomic configurations analysis. This structural homology coupled with nucleation barrier calculation was used to elucidate the non-classical nucleation pathway of BT2 crystallization: the similarity of the structural unit (Ti-O polyhedra) between the undercooled liquid and the metastable **β**-BT2 reduces the nucleation barrier and contributes to the preferential precipitation of **β**-like clusters. This work reveals the formation route of BT2 from cooling melt, which not only benefits the synthesis and application of this novel functional material but also provides a guideline of the crystallization process of titanates from melt at atomic level.

## Introduction

Most recently, a novel functional ceramic barium dititanate, BaTi_2_O_5_ (BT2), has drawn more and more attention due to its multifunctional properties and brilliant application prospect^1–3^. Distinct properties depend on a specific crystalline structure. The monoclinic phase (**γ**-BT2) is a high-temperature ferroelectric material^4^. The permittivity of **γ**-BT2 (ε_r_) can reach 25,000 in the vicinity of its Curie temperature (*T*_c_ = 743 K), and the piezoelectric coefficient, d_33_, is comparable with that of PbTiO_3_ according to a first principle calculation^5^. These excellent propertiess make **γ**-BT2 a promising lead-free ferroelectric candidate for ceramic capacitors^6^ and piezoelectric devices^7^. The metastable polymorphic phases of BT2 (**α**, **β**-BT2) can be observed during a continuous annealing process of glass^8^. These metastable phases consist of noncentrosymmetric polyhedra, which have a higher potential for yielding high dielectric, pyroelectric and nonlinear optical properties^9^. Although BT2 are expected to open a new vision in the BaO-TiO_2_ binary system, the crystallization mechanism of BT2 is a longstanding controversial issue and yet to be fully understood.

So far, the most widely used method to obtain bulk BT2 or doped BT2 material is from rapid cooling liquid^10–12^. However, the current knowledge about the crystallization mechanism of BT2 is still ambiguous. For example, O’Bryan and Thomsom regarded BT2 phase as a reaction intermediate in crystallizing from melt^13^. Kirby and Wechsler claimed that an liquid intermediate is required for nucleation of the BT2 phase^14^ without an in-depth explanation for the reason; West *et al*. proposed that the formation of BT2 is an example of the Ostward’s rule of successive reactions^15^, but they failed to demonstrate why the crystal embryo is stable near the liquidus temperature (~1663 K) that severiously deviated from BT2 thermodynamically stable temperature range (1493 K~1503 K). To clarify the nucleation and growth processes of BT2 from melt, the following critical questions should be answered: (i) whether BT2 crystals can form as the primary phase in melt beyond the thermodynamically stable temperature range; (ii) why BT2 is thermodynamically metastable but it can be obtained as the main phase rather than the thermodynamically stable phases such as BaTiO_3_ and Ba_6_Ti_17_O_40_ after melt quenching or rapid solidification. To solve the above problems, an insight into of the nucleation pathway of BT2 is essential. Here, undercooling solidification was used to investigate the nucleation pathway of BT2. It has the advantages of making the rapid crystallization process controllable, as well as providing valuable information on the initial stage of nucleation^16^.

Over the past few years, numerous direct or indirect research techniques have been developed to investigate the nucleation pathway, such as precipitation of minerals/protein from aqueous solution^17–19^, formation of colloidal crystals^20,21^, metal/alloys solidification^22,23^ and oxide materials crystallization^24,25^. Although the formation and evolution of nuclei and pre-nucleation clusters have been observed directedly through colloidal particales^21^ or HRTEM^17,18,23^, the indirect methods, such as heat events studies^26^, high-speed video camera^24^, microstructure studies^27,28^ and computational simulations^29,30^ are still the most widely used approaches for studying the nucleation path from extremely high temperature melt (especially undercooling melt). However, by these *ex situ* methods, it is diffcult to determine the crystalline phases and to identify the formation sequence simultaneously. Time-resolution *in situ* high energy X-ray diffraction (HEXRD) based on synchrotron radiation technology provides a new insight to reveal the nucleation pathway during extreme solidification process^31,32^. Therefore, a combination of undercooling solidification and HEXRD is expected to elucidate the nucleation pathway of BT2.

The purpose of studying the nucleation pathway is not only to determine the phases selections process but also to clarify the inherent law behind the sequence, which has long been a subject of research in solidification. Conventionally, the phases selections are manipulated by the nucleation barrier ${\rm{\Delta }}{G}_{n}^{\ast }$ or the growth kinetics, both of which are influenced by undercooling. With increasing undercooling, Ostwald’s step rule becomes valid due to the lower nucleation barrier of the metastable phases^33^. Moreover, the thermodynamically metastable phases with a high growth rate may also preferentially precipitate under specific undercooling conditions, for instance, the direct growth of the peritectic-phase NdBa_2_Cu_3_O_7-δ_^24^ and FeTi^28^ were observed in a supercooled melt below the peritectic temperature (T_P_). However, these classical interpretations are not sufficient to explain the crystallization process of oxide ceramics. Recent experimental studies have shown that there are several non-classical factors governing the nucleation of oxides from undercooled melt. Kuribayashi and Vijava Kumar made comprehensive investigation on the crystallization process of the rare-earth orthoferrite ReFeO_3_ (Re: La, Sm, Dy, Y, Yb, and Lu)^25,34^, and multiferroic phases ReMnO_3_ (Re: Rare-Earth Element)^35^. They proposed that the destabilized crystallography of the stable phase is the critical factor for the formation of metastable phases. The phases selection observed in rapid solidification process of Al_2_O_3_^36^, Al_2_O_3_-R_2_O_3_ (R: La, Er, Y)^37^ demonstrated that the structure of the undercooling melt^38,39^ and the low density liquid (LDL) generated from liquid-liquid phase transition^40,41^ could affect the nucleation pathway during undercooling solidification. The above studies suggest that the interrelation of crystal and liquid structure being the key to the nucleation process of oxides. Therefore, tracking the structural evolution from liquid to crystal and coupling it with the classical thermodynamic/kinetic properties may give a deep understanding of the nucleation pathway of oxides crystallization.

The present work aims to clarify and understand the crystallization process of BT2 from melt. Heat events, *in situ* HEXRD and microstructure studies are used to reveal the nucleation pathway. A polymorphic transition is found in BT2 crystallizing from undercooling melt. The transition follows a non-classical nucleation sequence: undercooled melt → **β**-BT2 → **γ**-BT2. To understand the mechanism behind this two-step nucleation pathway, structural parameters of liquid are tracked from above the liquidus to the undercooling state, and the sequence of nucleation is disscussed based on the degree of structural dissimilarity between the undercooled liquid and the corresponding polymorphic phases. This work is expected to resolve the dispute about BT2 forming from cooling melt and to provide a convenient approach to prepare the ferroelectric **γ**-BT2 and the metastable phase (**β**-BT2).

## Methods

### Sample preparation and triggering nucleation

**Figure 1:**
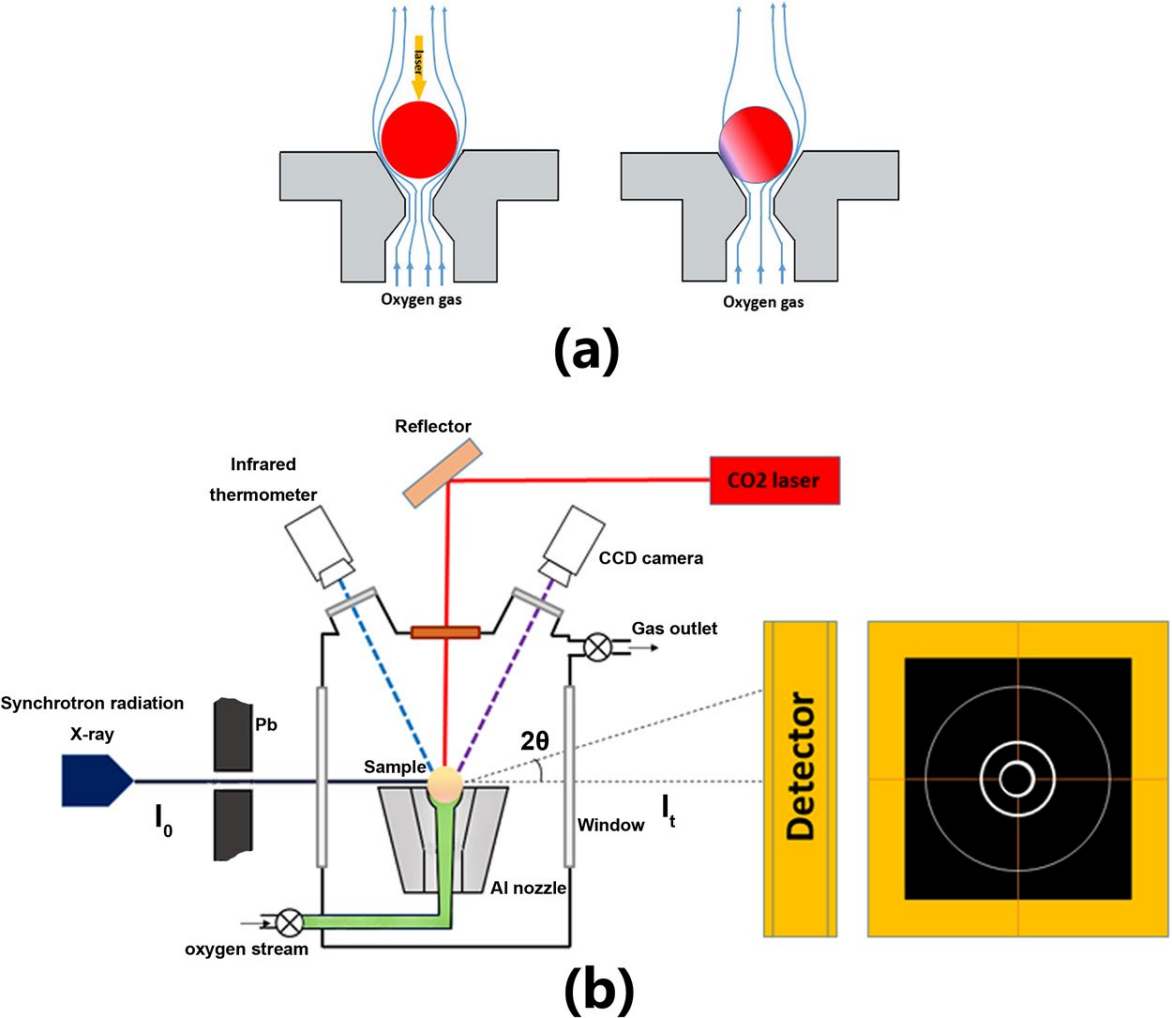
The schematic of experiments: (**a**) Triggering nucleation process by decreasing the air flow. (**b**) The aerodynamic levitation device for HEXRD experiments at the SSRF station BL13W1.

Highly pure BaCO_3_ (99.95%, Aladdin, China), TiO_2_ (99.99%, Aladdin, China) powders were used as starting materials. BaCO_3_ and TiO_2_ were stoichiometrically weighed according to the  chemical formula of BaTi_2_O_5_ and mixed in an agate mortar for 2 h with a small amount ethanol addition. The mixed powders were pressed into pellets of 20 mm in diameter and 1~3 mm in thickness under a pressure of 20 MPa. These pellets were put into a muffle furnace to sinter at 1273 K for 12 h. Subsequently, a containerless processing was carried out by a ADL furnace. The spherical droplet was suspended by controllable pure oxygen flow and heated by a 100 W CO_2_ laser. CCD camera was used to monitor the melting and cooling processes, and a optical pyrometer with wavelength at 1.255 μm was applied for recording the temperature. The triggering nucleartion process was achieved by gradually decreasing the oxygen flow, which finally destroyed the stable levitation and induced a slight contact between the undercooled sample and the wall of nozzle, as shown in Fig. 1(a). During this process, the output power of laser was manipulated to ensure stable temperature and desired undercoolings, and once recalescence took place, the laser was shut down immediately.

### HEXRD experiment

The HEXRD experiment was carried out by incorporating the ADL facilities into beam line station BL13W1 of Shanghai Synchrotron Radiation Facility (SSRF), as shown in Fig. 1(b). ADL furnace was assembled on a two-axis translation stage to ensure precise alignment of the specimen with the synchrotron radiation X-ray beam. The high energy X-ray with an energy of 69.525 KeV (calibrated by tungsten target) and a wavelength of 0.017835 nm provides a sufficient scattering range (Q) and reduces the effects of absorption and multiple scattering. Diffraction signal was collected by a Silicon flat-panel X-ray image plate (XRD 1621 AN3 ES). The X-ray beam was partially intercepted by the top of the specimen, which was suitable for high angle diffraction.

### Data processing

In the HEXRD experiment, the initial diffracted intensity, *I*_*s*, *a*_(*Q*), was extracted by integrating the image plate data through software fit2D. The scattering intensities of the kapton film and air *I*_*a*_(*Q*), obtained in the scattering measurement without samples, were subtracted from the initial diffracted intensities. Before the calculation of the structure factor S(Q), the raw data was corrected for polarization, absorbtion, multiple scattering, Compton scattering and fluorescence. As the X-ray conducted on a spherical surface rather than a conventional plane, direct volume integration method was used to determine the attenuation coefficients and the multiple scattering. The total x-ray structure factor S(Q) can be derivered from the normalized x-ray scattering intensity, *I*_s_(*Q*):

1 $${\rm{S}}({\rm{Q}})-1=[\beta {I}_{{\rm{s}}}(Q)-{\sum }_{{\rm{i}}}{c}_{{\rm{i}}}\,{f}_{{\rm{i}}}^{2}(Q)-{I}_{{\rm{inc}}}(Q)-{I}_{{\rm{ms}}}(Q)]{[{\sum }_{{\rm{i}}}{c}_{{\rm{i}}}{f}_{{\rm{i}}}(Q)]}^{-2}$$

Where *c*_i_ is the atomic fraction of element i, *f*_i_(*Q*) is the atomic X-ray form factor of element i, *I*_inc_(*Q*) is the intensity of compton scattering, *I*_ms_(*Q*) is the intensity of multiple scattering that is mainly based on double multiple scattering. ${\rm{Q}}=4{\rm{\pi }}\,\sin ({\rm{\theta }})/{\rm{\lambda }}$ is related to the scattering angle 2θ and the x ray wavelength λ. β is the normalization factors, which is calculated by Korgh-Moe-Norman method.

2 $${\rm{\beta }}=\frac{{\int }_{0}^{{Q}_{max}}{Q}^{2}\frac{{\sum }_{{\rm{i}}}{c}_{{\rm{i}}}\,{f}_{{\rm{i}}}^{2}(Q)+{I}_{{\rm{inc}}}(Q)+{I}_{{\rm{ms}}}(Q)}{{[{\sum }_{{\rm{i}}}{c}_{{\rm{i}}}{f}_{{\rm{i}}}(Q)]}^{-2}}\exp (-\gamma {Q}^{2}){\rm{dQ}}-2{\pi }^{2}{\rho }_{0}}{{\int }_{0}^{{Q}_{{\rm{\max }}}}{Q}^{2}\frac{{I}_{{\rm{s}}}(Q)}{{[{\sum }_{{\rm{i}}}{c}_{{\rm{i}}}{f}_{{\rm{i}}}(Q)]}^{-2}}\exp (-\gamma {Q}^{2}){\rm{dQ}}}$$

where the γ is a convergence factor, ρ_0_ is the atomic number density, which is calculated by

$${\rho }_{0}=\rho {{{\rm N}}}_{0}/{\sum }_{i}{c}_{i}{M}_{i}\times {10}^{-24}({\AA }^{-3})$$

The total correlation function T(r) can be obtained by sine Fourier transforming from total structure factor S(Q)-1.

3 $${\rm{T}}({\rm{r}})=4\pi \rho r+\frac{2}{\pi }{\int }_{0}^{{Q}_{max}}Q[S(Q)-1]L(Q)\sin (rQ)dQ$$

where r is the interatomic distance, *L*(Q) is the Lorch modification function^42^ to reduce the truncation effect of the integral which is used in practice.

### Reverse monte carlo (RMC) simulation

The RMC simulation was performed using a RMC_POT++ code^43,44^. The starting configuration consisting of 8000 atoms was created by hard sphere Monte Carlo (HSMC) simulation. Several types of constrains were added: the atom-atom approaches, Ti-O connectivity (all titanium atoms were coordinated to certain number oxygen atoms up to 2.5 Å) and interatomic potential between Ti-O, Ba-O and O-O pairs. The choice of these constrains were determined to avoid physically unrealistic structures. After the HSMC simulation, several RMC simulations containing S(Q), T(r) data were performed. The structural information is derived from counting the atomic configurations generated by fifteen independent RMC simulations.

### Materials characterizations

The phases were identified by Cu Kα X-ray diffraction (XRD, Rigaku Ultima IV). Before measurements, all the spherical samples were ground into powder. The morphology was characterized by scanning electron microscopy (SEM, JEOL JSM-7800F Prime). The spherical sample was ground and polished to flat surface and subjected to thermal etch and gold sputtering before SEM observations. Raman spectroscopy was obtained by confocal laser Raman spectrometer (LabRAM HR Evolution) excited by a 532-nm laser. The diameter of the laser spot is ~1 μm. The thermodynamic properties were analyzed by differential scanning calorimetry (DSC, Netzsch STA449 F3), where a pair of platinum crucibles were used to melt BT2. The testing temperature range was from room temperature to 1500 °C with a heating rate of 10 K min^−1^ in an atmosphere of 21 vol.% oxygen and 79 vol.% argon.

## Results

### Thermodynamic properties and the critical undercooling

Hypercooled solidification gives insight into the primary crystallization processes, because the primary morphology and solidified phases will be remained under hypercooled condition^45^. Therefore, the critical undercooling of hypercooled solidification is an important parameter for studying undercooling solidification. As for hypoeutectic composition like BT2, the hypercooling limit can be calculated by:

4 $${\rm{\Delta }}{T}_{{\rm{hyp}}}={\rm{\Delta }}{H}_{{\rm{f}}}/{C}_{{\rm{p}}}^{{\rm{L}}}+({T}_{{\rm{L}}}-{T}_{{\rm{S}}})$$

where Δ*H*_f_ is the enthalpy of BT2 fusion, ${C}_{{\rm{p}}}^{{\rm{L}}}$ is the specific heat of the undercooled melt. However, it is troubling that these basic thermodynamic parameters of BT2 are unavailable.

**Figure 2:**
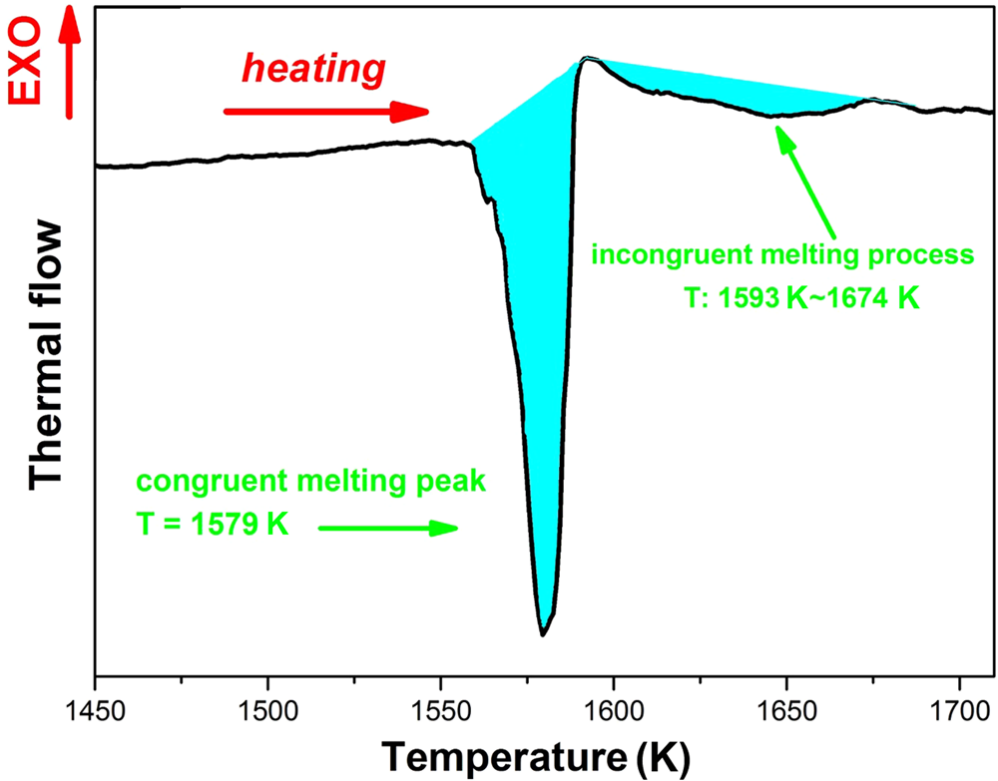
DSC pattern of BT2 melting process, the melting enthalpy is calculated by integral of peaks.

The enthalpy of fusion can be measured by DSC and the results are shown in Fig. 2. During BT2 melting process, a congruent melting peak related to solidus and an incongruent melting peak within the temperature range from solidus to liquidus are observed from DSC pattern. The enthalpy of fusion can be estimated at 81.4 KJ mol^−1^ by the integral area surrounded by the endothermic peak and the baseline. The mixing enthalpy of both BaTiO_3_ (BT) and BT2 are far less than the prediction by Roult’s rule, which may be attributed to a strong interaction between BaO and TiO_2_.

The specific heat of undercooled liquid BT2 was calculated from the cooling curves obtained by blocking the laser, using the same method proposed by Nagashio and Kuribayashi^46^. When the heat source is removed, the extreme cooling process of levitated sample is mainly regulated by heat convection and heat radiation, thus the temperature can be described by the following equation.

5 $$\frac{d{T}_{{\rm{s}}}}{dt}=-\frac{3}{{c}_{{\rm{s}}}{\rho }_{{\rm{s}}}R}[h({T}_{{\rm{s}}}-{T}_{0})+\sigma \varepsilon ({T}_{{\rm{s}}}^{4}-{T}_{0}^{4})]$$

As a result, the specific heat can be calculated if the emissivity *ε* and the heat-transfer coefficient *h* are obtained. In this work, *ε* was calibrated to 0.9 referred from BaTiO_3_ measured data^47^ and *h* could be calculated by Whittaker equation^48^.

6 $$(0.4{{\rm{Re}}}^{\frac{1}{2}}+0.06R{e}^{\frac{2}{3}})P{r}^{0.4}{(\frac{{\eta }_{{\rm{s}}}}{{\eta }_{0}})}^{\frac{1}{4}}=\frac{2hR}{\lambda }$$

As the average velocity of the oxygen flow around the sample is the key to solving the Whittaker equation, the stable suspension condition must be considered. The gravitation of sample should be equal with the sum of aerodynamic drag and buoyancy, which satisfied the following equation:

7 $$\frac{4}{3}\pi {R}^{3}{\rho }_{{\rm{s}}}g=\frac{1}{2}{C}_{{\rm{D}}}\pi {R}^{2}{\rho }_{{\rm{o}}}{\nu }^{2}+\frac{4}{3}\pi {R}^{3}{\rho }_{{\rm{o}}}g$$

*C*_D_ is the drag coefficient, which is related to the Reynolds number.

8 $${C}_{{\rm{D}}}={(0.55+\frac{4.8}{\sqrt{Re}})}^{2}10 < Re < {10}^{4}\,$$

**Figure 3:**
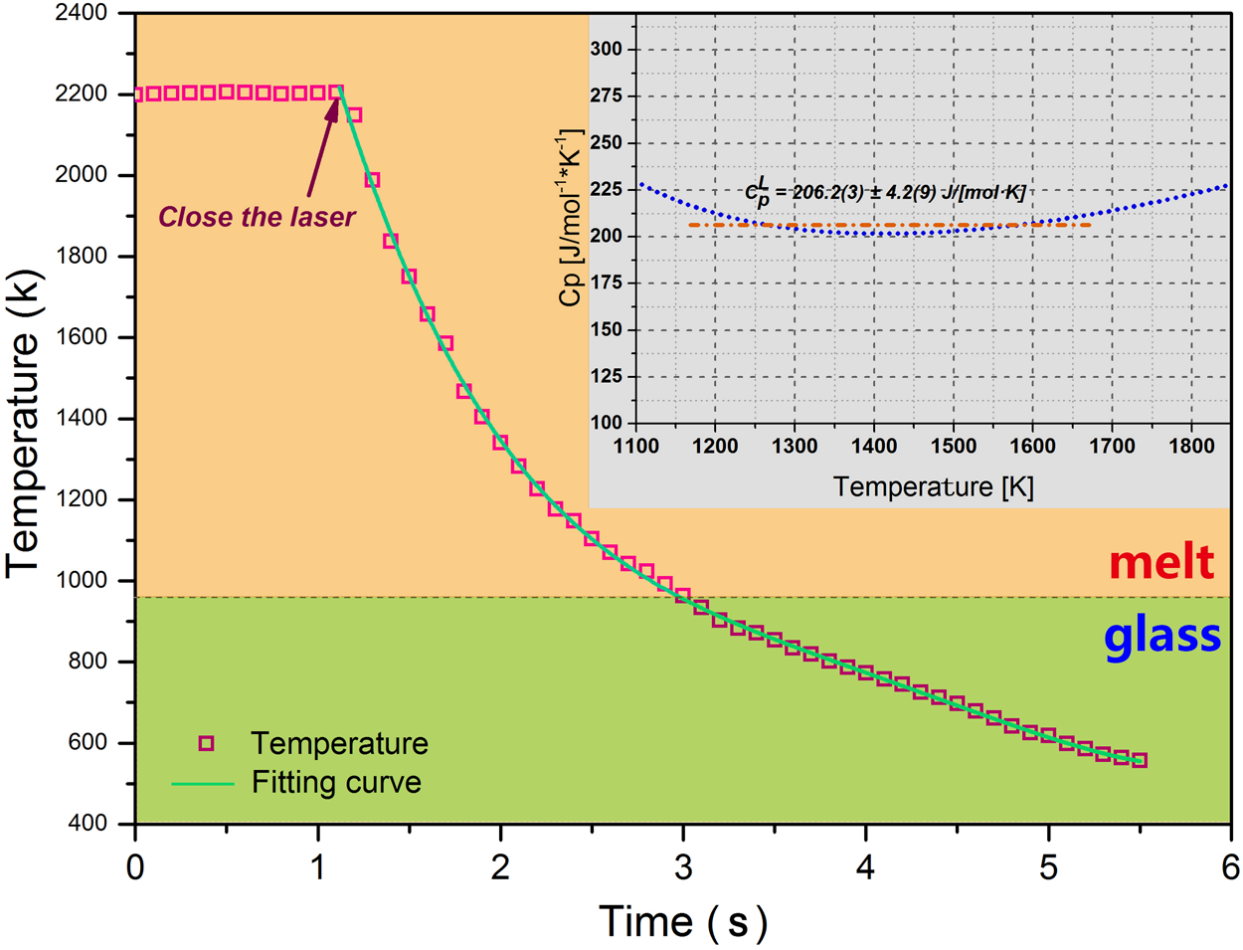
Thermal profile of extreme cooling process after blocking the CO_2_ laser. Inset is the calculated specific heat of BT2 melt in the temperature range of 1163 K–1663 K.

The relevant cooling curve is shown in Fig. 3, the cooling rate $\frac{d{T}_{{\rm{s}}}}{dt}$ can be obtained by taking a derivative of the cooling curve. The meaning and value of the physical parameters mentioned in Eqs (5–8) are listed in Table 1. Based on the above, the specific heat ${{\rm{C}}}_{p}^{L}$ of BT2 melt can be obtained. As shown in the inset figure of Fig. 3, the value of ${{\rm{C}}}_{p}^{L}$ is 206.2 ± 4.3 Jmol^−1^ K^−1^, with a small uncertainty of 2.1%, in the temperature range from 1163 K to 1663 K.

**Table 1:** Physical parameters used in the calculation of melt specific heat and hypercooling limit of BT2.

Property	Symbol [unit]	Value
Atmosphere temperature	*T*_0_ [K]	298
Dynamic viscosity of oxygen	*η* [Pa·S]	3.08 × 10^−5 ^^46^
Density of oxygen	*ρ*_0_ [g L^−1^]	1.325
Specific heat of oxygen	*C*_p_ [J g^−1^ K^−1^]	0.916^46^
Thermal conductivity of oxygen	*λ* [W m^−1^K^−1^]	0.0399^46^
Radius of sphere	*R* [m]	0.000889
Density of sphere	*ρ*_s_ [g mL^−1^]	3.945
Emissivity	*ε*	0.9^47^
Stefan–Boltzmann constant	*σ* [W m^−2^ K^−4^]	5.67 × 10^−8^
Mean speed of Oxygen surrounding sample	*υ* [m s^−1^]	15.82
Reynolds number	Re	1180
Planck number	Pr	0.7057
Heat-transfer coefficient	*h* [W m^−2^ K^−1^]	283.8
Specific heat of BT2 melt	*C*_p_^L^ [J mol^−1^ K^−1^]	206.2(3) ± 4.2(9)
Heat of fusion	Δ*H*_f_ [KJ mol^−1^]	81.4

By using the obtained Δ*H*_f_ and ${{\rm{C}}}_{p}^{L}$ in Eq. (4), the value of hypercooling limit Δ*T*_hyp_ was calculated as 459.8 K, which should be further identified in the following triggering solidification.

### Triggering nucleation solidification

**Figure 4:**
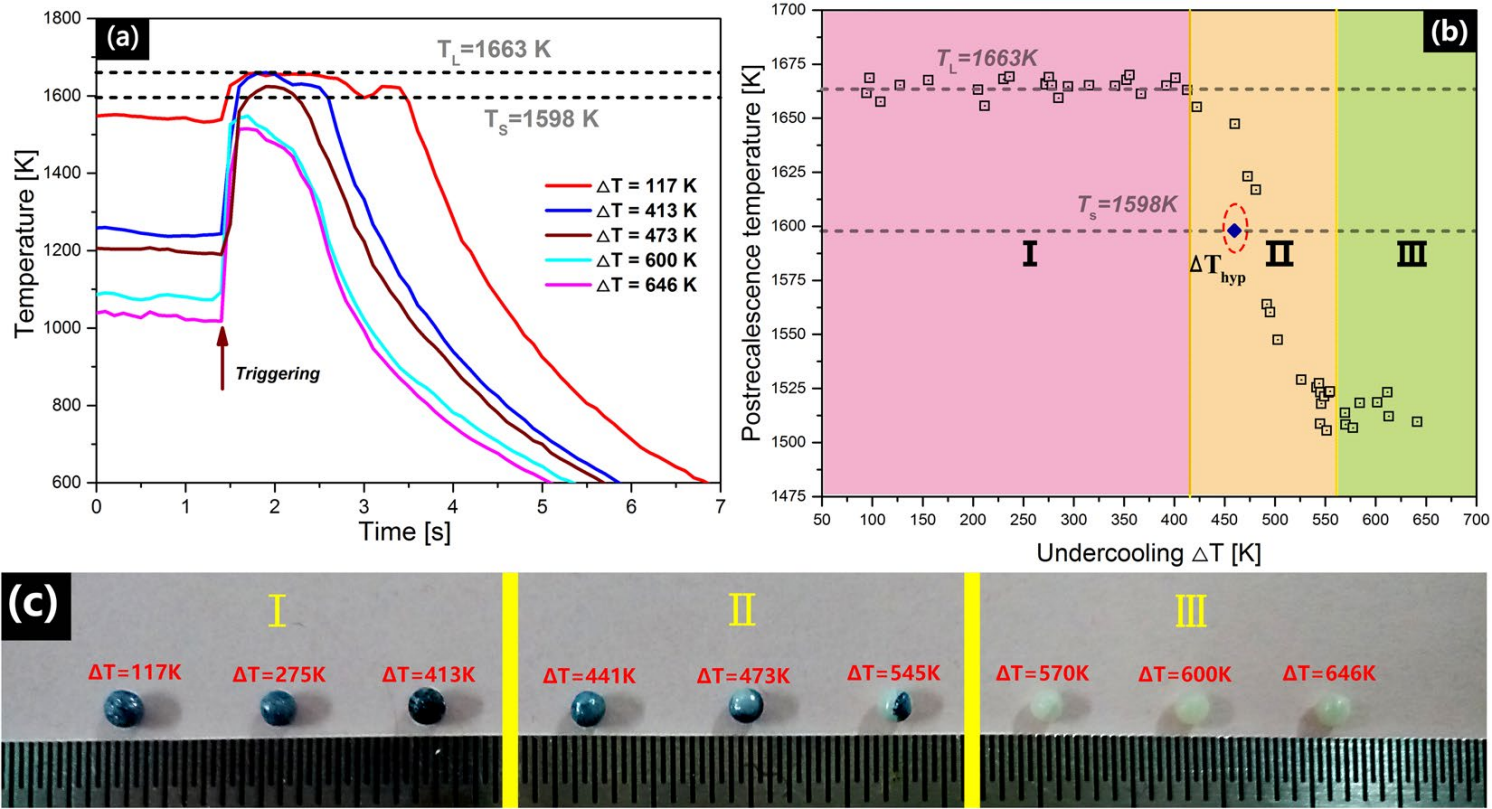
(**a**) Typical cooling curves obtained under different undercoolings after being triggered. The liquidus and solidus are shown by dash lines. (**b**) Relationship between undercooling and post-recalescence temperature, where three characteristic sections were found and highlighted as I, II, III. (**c**) Photograph of samples fabricated under different undercooling temperatures, and they were distinguished by the evolution of samples color from dark blue to combined dark blue and white and finally to pure white.

All samples were heated to and remained at 2100 K for ~30 s. This superheated treatment not only ensured the complete melting but also passivated heterogeneous nuclei to obtain high undercooling. The samples were then slowly cooled to desired undercooling before being triggered nucleation. The cooling curves after being triggered under different undercoolings are shown in Fig. 4(a). The temperature increased dramatically once nucleation occurred because the latent heat of solidification rapidly released during nucleation and crystal growth. When nucleated under a small undercooling (Δ*T* = 117 K), the post-recalescence temperature of the sample increased to near the liquidus and presented an obvious recalescence platform. With increaseing undercooling, the recalescence platform became shorter and eventually disappeared at 473 K. The maximum undercoooling obtained was 646 K, corresponding the minimum post-recalescence temperature less than solidus 80 K, which indicated hypercooled solidification was fulfilled.

The relationship between the post-recalescence temperature and undercooling is shown in Fig. 4(b). Three different stages were observed, demarcated by two anomalous turning points at undercoolings of approximately 413 K and 558 K. The turning points may be attributed to changes in the heat release or emissivity of the solidified product. The macroscale appearance of the samples is shown in Fig. 4(c). The hypercooling limit identified by this triggering solidification is ~475 K, which is very close to the caculated result 459.8 K, indicating the validity of the above evaluation of the thermodynamic properties of BT2.

As shown by Fig. 4(c), all the samples are spherical with a diameter of ~2 mm. The samples prepared at small undercoolings are dark blue in color, which may be related to the presence of oxygen vacancies. With increasing undercooling, color of the samples changes from dark blue (I), to a combination of dark blue and white (II) to bright white (III). As oxygen vacancies can be generated more easily at high undercoolings, it is reasonable to attribute the color change to a phase transition rather than the disappearance of oxygen vacancies.

**Figure 5:**
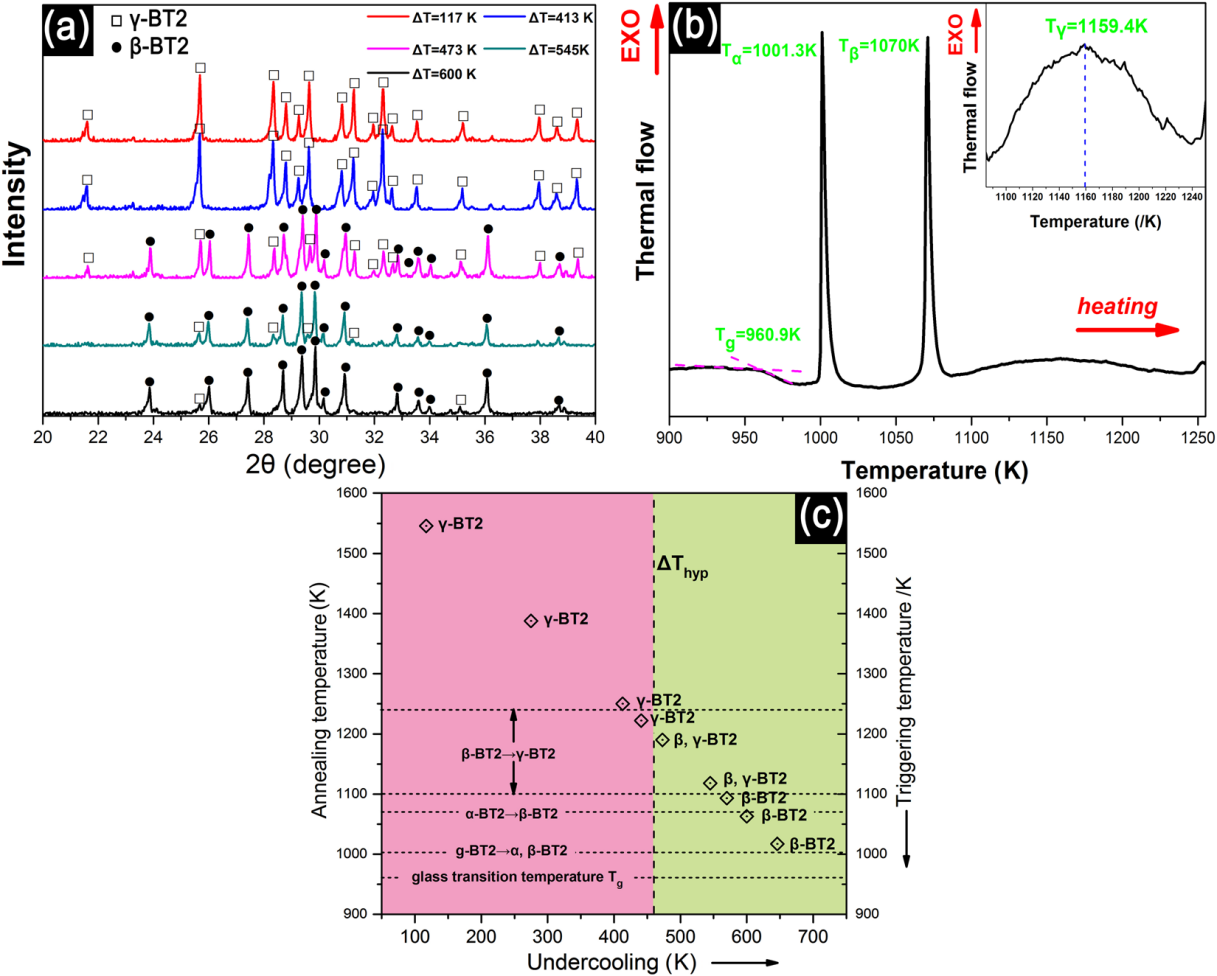
(**a**) XRD spectra of samples obtained by triggering at different undercoolings. (**b**) The DSC pattern of BT2 glass annealing process. (**c**) The relationship of triggering nucleation temperature (undercooling) to solidified phase composition. Horizontal dotted lines indicate the phase transition temperature during glass annealing.

In order to verify the hypothesis, five representative samples solidified under different undercoolings were powdered for XRD, and the results are presented in Fig. 5(a). γ-BT2 phase is observed in the dark blue samples parepared under undercoolings of 117 K and 413 K, while almost pure **β**-BT2 is observed in the bright white sample under an undercooling of 600 K. In the sample showing a combination of dark blue and white obtained under medium undercoolings of 473 K and 545 K, both **γ** and **β** phases are detected. In all samples, no other phase is detected, indicating that BT2 nucleates directly from the melt rather than from a reaction intermediate. Moreover, the evolution of phase indicates a polymorphic transition occuring in BT2 undercooling solidification, which is analogous to the annealing process of BT2 glass (**g**-BT2)^8^. Figure 5(b) presents the DSC profile of the g-BT2 during heat treatment in the temperature range from 900 to 1250 K, where the glass transition temperature is 960.9 K, and the two sharp exothermic peaks correspond to two phase transitions of **g**-BT2 to **α**, **β**-BT2 (at 1001.3 K) and **α**-BT2 to **β**-BT2 (at 1070 K), respectively. The phase transition from **β**-BT2 to **γ**-BT2 shows a weak exothermic peak within a relatively wide temperature range of 1100–1220 K with a peak temperature about 1159.4 K, as shown in the inset of Fig. 5(b). The phase composition as a function of undercooling is shown in Fig. 5(c), where the phase transition temperatures during **g**-BT2 annealing are also shown for comparison. An interesting relationship between the phase composition and undercooling (triggering nucleation temperatures) can be observed: the **γ**-BT2 phase is formed at small undercooling, and **β**-BT2 can be obtained during hypercooled solidification. The phase transition (**β**-BT2 → **γ**-BT2) is estimated to occur over an undercooling range of 473–570 K, corresponding to a nucleation temperature range of 1093–1190 K, which lies in the solid-state phase transition temperature range (1100–1240 K) during the glass annealing process. The above result indicates that the solid-state transition may play a role in the polymorphic transition during undercooling solidification.

Based on the above experimental results, two possible nucleation pathways are deduced for BT2 undercooling solidification: (1) a **γ**-phase nucleus forms at low undercoolings, and the **β** phase nucleates at large enough undercoolings; (2) **β** phase always nucleates from the undercooled melt if the melt temperature is higher than the phase transition temperature (**β**-BT2 → **γ**-BT2), and **γ**-BT2 nucleates as a result of the solid-state phase transition.

### Determination of the nucleation pathway

**Figure 6:**
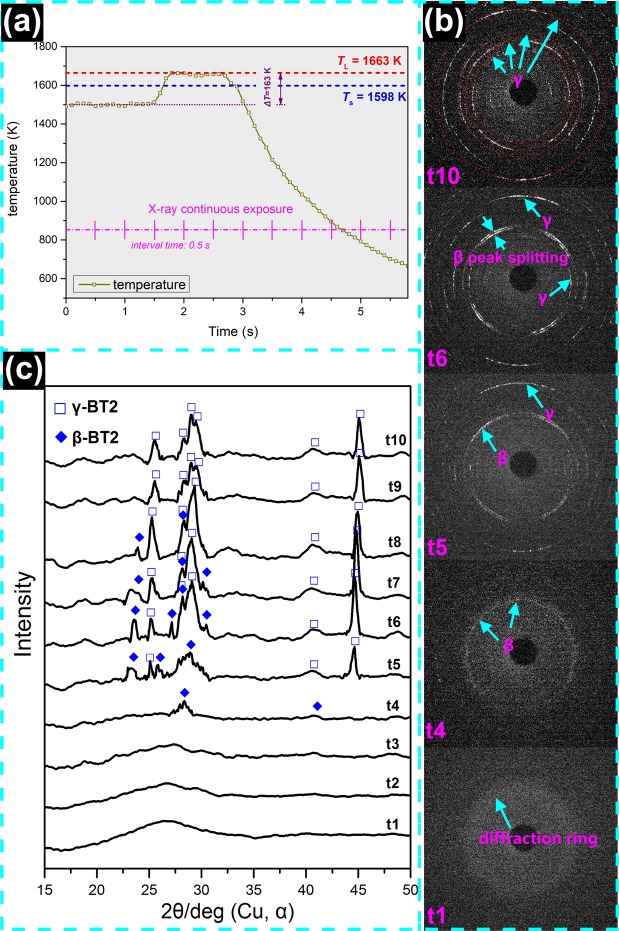
*In situ* HEXRD result of the sample that was triggered at Δ*T* = 163 K. (**a**) Temperature-time profile of the continuous exposure experiment; (**b**) two-dimensional diffraction patterns captured by PE panel with 0.5 s exposure time. (**c**) The integrated HEXRD diffraction pattern showing the solidification path during this undercooled solidification process. These patterns are stacked from bottom to top in sequence of elapsing time. In order to better refer to powder diffraction data as shown in Fig. 5(a), the diffraction angle obtained by synchrotron radiation X-ray (wavelength *λ* = 0.017199 nm) was transformed into standard Cu-target X-ray (wavelength *λ* = 0.15406 nm) diffraction angle by using formula:$\,\frac{{\sin {\rm{\theta }}}_{1}}{{{\rm{\lambda }}}_{1}}=\frac{{\sin {\rm{\theta }}}_{2}}{{{\rm{\lambda }}}_{2}}$.

*In situ* real time HEXRD was employed to identify the most possible nucleation pathway during the crystallization process of BT2. A continuous exposure experiment was conducted to verify whether **γ**-BT2 directly nucleates at small undercooling (Δ*T* = 163 K). The temperature-time profile is illustrated in Fig. 6(a). A series of representative two-dimensional diffraction images  are shown in Fig. 6(b). Before nucleation, the undercooled melt presents as diffraction rings (t1). When nucleation and recalescence occur, a few diffraction spots can be detected around the diffraction rings of the melt (t4). Polycrystalline diffraction rings are not observed in the recalescence stage possibly because crystallization commence with only a few nuclei. Over time, the concentric polycrystalline diffraction rings appear due to multiplication of the primary crystalline phase or appearance of a novel nucleation process during continuous crystallization process (t5 - t10). Figure 6(c) shows the integrated diffraction patterns over time. Before crystallization, typical diffuse scattering peaks are observed in t1 to t3, corresponding to the diffraction rings in the two-dimensional diffraction images for melt. After nucleation, sharp crystalline diffraction peaks can be observed from t4 to t10. Combining the above HEXRD results with the powder XRD patterns of **γ-** and **β**-BT2 (Fig. 5(a)), it is found that the **β**-BT2 diffraction peaks are prominent at the early stage of crystallization (t4 and t5), indicating that **β**-BT2 is the primary phase even at small undercoolings. Over time, two interesting features are observed from t6 to t8: (i) more refined **β**-BT2 diffraction peaks appear, corresponding to the growth and multiplication of the primary **β** phase; and (ii) some **β**-BT2 diffraction peaks disappear or split into **γ**-BT2, suggesting that the primary **β** phase gradually transformed into **γ** phase during the crystal growth process. In the final stage (t9 and t10), almost all the peaks can be indexed to **γ**-BT2.

Based on the above results and analysis, it is concluded that the most likely nucleation pathway in BT2 undercooling solidification is melt → **β**-BT2 → **γ**-BT2. The first transition is the nucleation of the **β** phase from undercooling liquid, followed by a solid-state phase transition from **β**-BT2 to **γ**-BT2 during crystal growth process when the liquid temperature is sufficiently high.

### Evolution of microstructure

**Figure 7:**
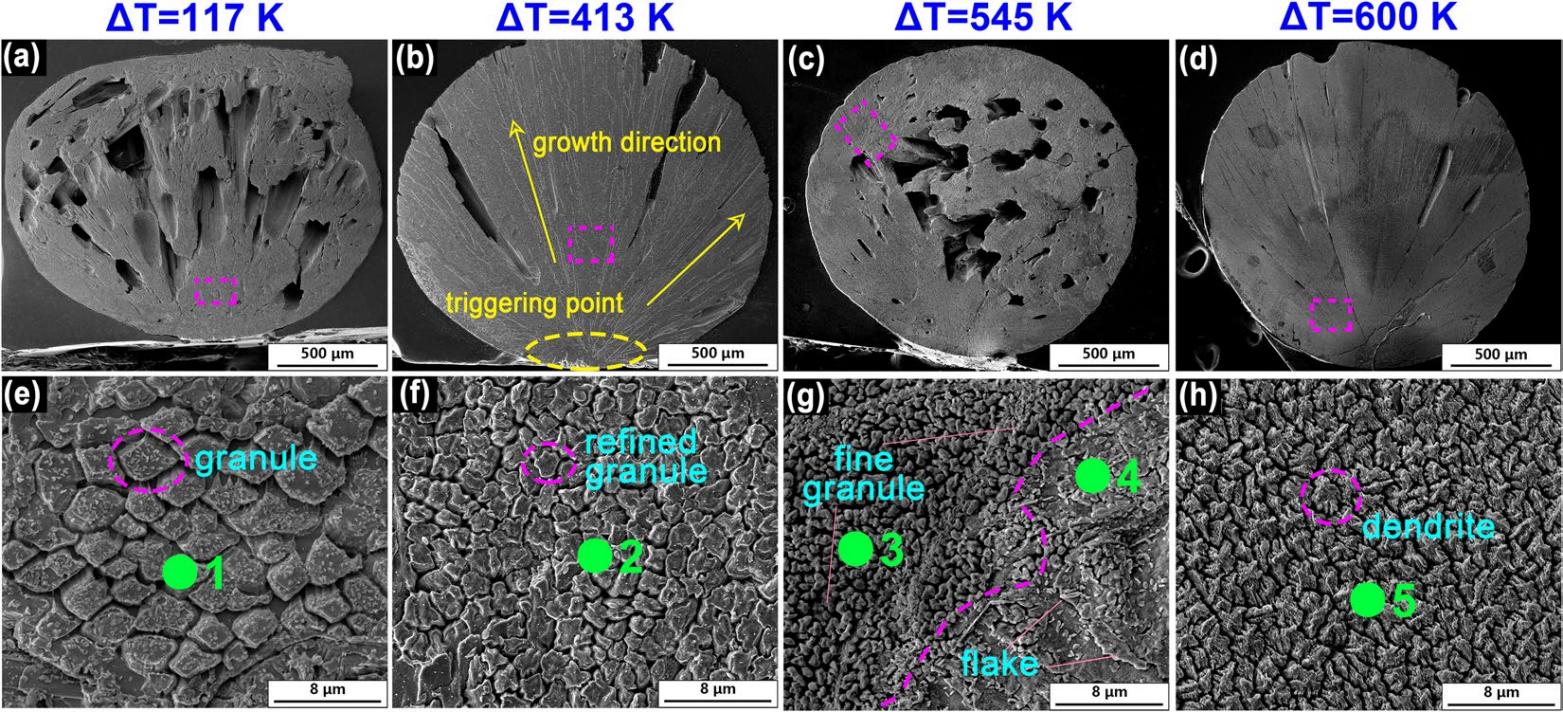
SEM images of samples obtained at different undercoolings: (**a**,**e**) morphology obtained at Δ*T* = 117 K; (**b**,**f**) morphology obtained at Δ*T* = 413 K; (**c**,**g**) morphology of the hybrid microstructure, which corresponds to the part dark blue and part white sample (Fig. 4(c)), obtained at Δ*T* = 545 K; and (**d**,**h**) the morphology obtained at a high undercooling of Δ*T* = 117 K. The green dot and number in (**e**–**h**) indicate the position for Raman spectroscopy measurements.

**Figure 8:**
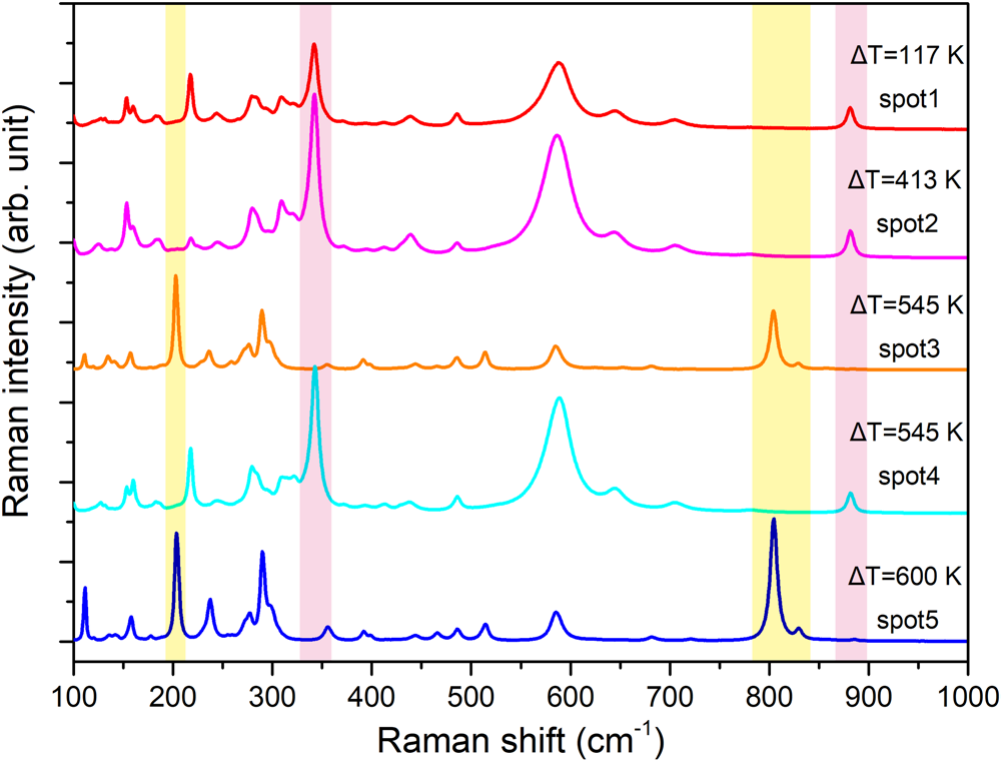
Raman spectra of samples obtained under different undercoolings, spot1 to spot5 corresponds to the region marked in Fig. 7(e–h). Canary yellow bright area represents **β**-BT2 Raman peaks of 203.9 cm^−1^ and 804.2 cm^−1^, pink bright area represents **γ**-BT2 Raman peaks of 342.3 cm^−1^ and 881.5 cm^−1^.

Figure 7 shows the longitudinal microstructures of BT2 obtained at different undercoolings. All samples show directional growth from the nucleation point to the boundary of the sample (Fig. 7(a–d)). At small undercooling (Δ*T* = 117 K), as shown in Fig. 7(a,e), large volume shrinkage during crystallization results in many shrinkage cavities. Polygonal grains with obvious growth edges are observed, which suggests a typical faceted growth. At a medium undercooling (Δ*T* = 413 K), the grains are refined, and the boundary becomes more circular, as shown in Fig. 7(b,f). Furthermore, when the undercooling reaches 545 K, a hybrid microstructure is obtained, Fig. 7(c,g). The hybrid microstructure has a distinct dividing line resulted from the mixture of solidified phases, which is further characterized by Raman spectroscopy. As shown in Fig. 8, Spot3 consists of the same Raman peaks with spot 5 (**β**-BT2), however, spot4 consists of the same Raman peaks with spot1, 2 (**γ**-BT2). Therefore, from microstructure point of view, **β**-BT2 presents as irregular fine gains and **γ**-BT2 presents as irregular flakes, which indicates that the crystal growth mode of the two phases is different at Δ*T* = 545 K. Furthermore, an obvious regional distribution, rather than coupled growth, also suggests that the polymorphic transition originates from a stepping nucleation instead of a competitive growth. Unexpectedly, at an undercooling of 600 K (Fig. 7(d,h)), faceted dendrites are observed, indicating an obvious anisotropic growth, which is rarely observed in metals and alloys.

**Figure 9:**
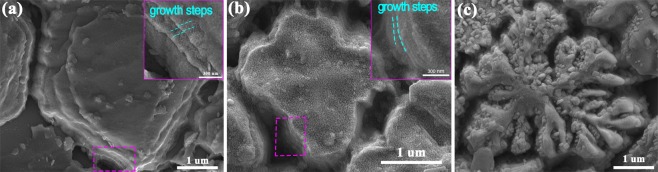
High-resolution SEM images of the crystals under different undercoolings: (**a**) 117 K, (**b**) 413 K, and (**c**) 600 K. The insets of (**a**,**b**) show the growth steps on the crystal boundary. The regions selected are marked in Fig. 6(c,f,h) by magenta ellipse.

High-magnification images of the three typical crystals highlighted by magenta ellipses in Fig. 7(e,f,h) are presented in Fig. 9(a–c). Obvious growth steps can be observed on the edge of the grains obtained under undercoolings of 117 K and 413 K, which suggests that the crystals grow in a lateral growth mode. Comparing the two, at Δ*T* = 413 K, the grains are refined, the grain boundaries transform from straight to circular, and the growth steps are narrow and compact. When the undercooling increases to 600 K, Fig. 9(c), snowflake-like dendrites consisting of many irregular granules and embossments are obtained. The surficial pattern of grains crystallized from undercooling melt is obviously distinct from that precipitated from solution^18^, which suggests that the nucleation and growth of crystals are not identical in these two cases.

## Discussion

A non-classical two step nucleation pathway during BT2 crystallization is identified: metastable **β**-BT2 phase always nucleates first from undercooling liquid, subsequently, a polymorphic transition caused by solid-state phase transition determines the final phase composition. The preferential nucleation of **β**-BT2 indicates that this metastable phase possesses a strong kinetic stability in undercooling melt. In this section, the structural origins of this non-classical nucleation pathway are discussed, and the nucleation barrier of the two crystallized phases are further calculated to reveal the kinetic stability of **β**-BT2.

The relationship between the structure of undercooling liquid and nucleation is a hot issue. In previous studies, scattering experiments and molecular dynamics simulations elucidate that the evolution of undercooling liquid plays an important role in arranging the nucleation pathway of metals^49,50^, colloids^20,51^ and polymers^52^. However, related work on oxide ceramics is rarely reported. One reason is that the structure of oxide is not readily available due to its high melting point and high corrodibility to container. Besides, the structure of oxides crystalline phases are relatively lower symmetry than that of spherical system (metal or colloid), which will complicate the research. In here, we tracked the structural evolution of BT2 undercooling liquid through HEXRD combined with containerless processing^53,54^. The three-dimensional atomic configurations are further created by using RMC simulation. The structural differences between undercooling melt and solidified phases are probed to provide a nucleation scenario of BT2.

**Figure 10:**
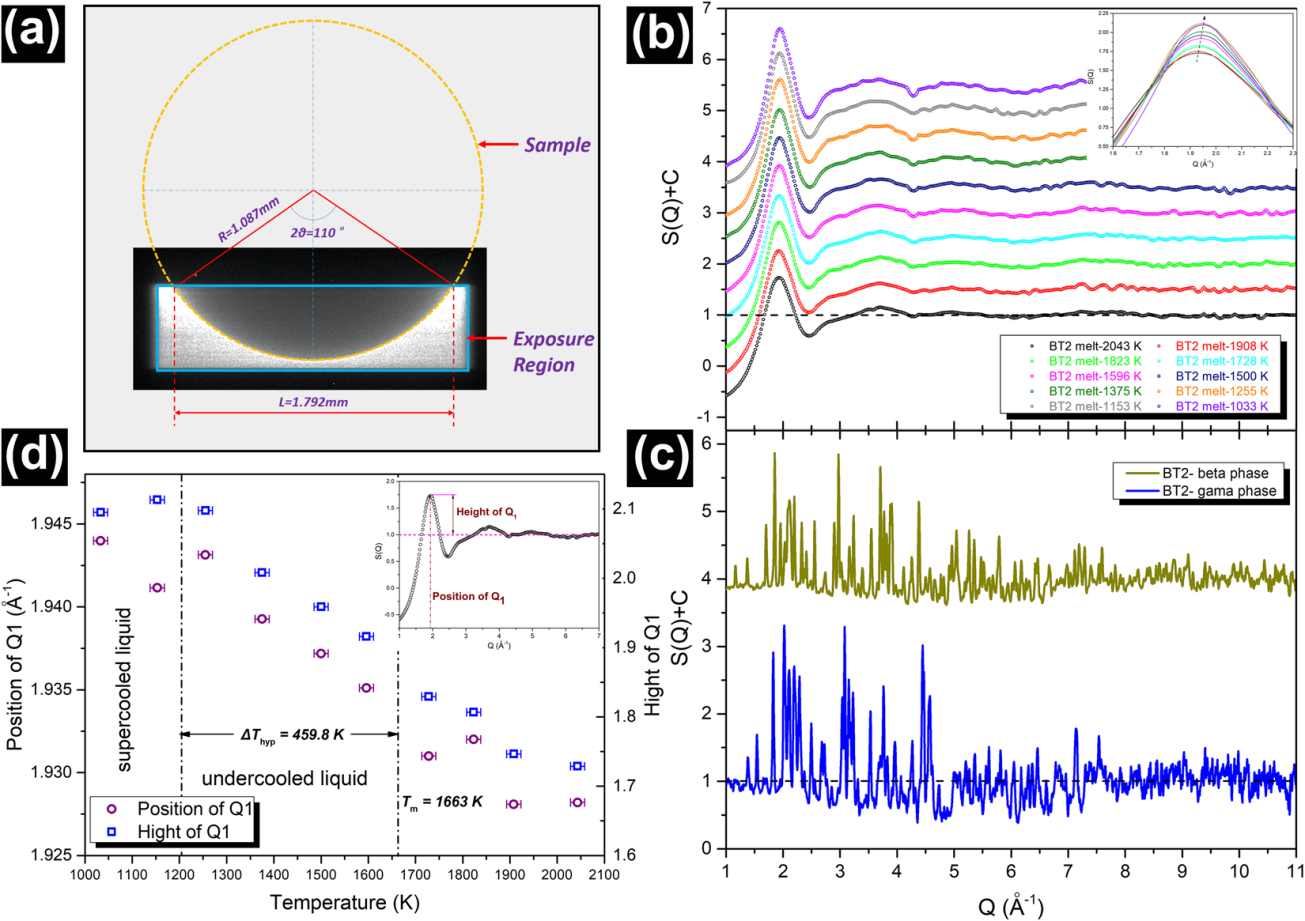
(**a**) The schematic of sample’s exposure region. (**b**) Structure factors for undercooled melt BT2 (from 2043 K to 1033 K), all the curves are offset a constant. The inset presents the amplification of the FSDP without any offset. (**c**) Structure factors for crystalline **γ**-BT2 and **β**-BT2. The samples were prepared by being triggered in the situation of undercooling at 120 K and 643 K respectively. (**d**) The relationship between the FSDP of BT2 melt structure factors and temperature.

Figure 10(a) illustrates the exposure region of a sample during the HEXRD scattering experiment, which provides the geometric parameters for the following data correction. Total structure factor S(Q) of the undercooling liquid and the crystalline phases of BT2 are shown in Fig. 10(b,c), respectively. Exposure time for all the samples is fixed at 60 s. S(Q) of the undercooling liquid exhibts four peaks between 1~10 Å^−1^ with a prominent first sharp diffraction peak (FSDP) around 2 Å^−1^, which is a discernible feature in glass-forming liquids. The local amplification of FSDP is shown by the inset figure in Fig. 10(b), a real asymmetry indicates uneven bondings in the structural units. Comparing the total structure factors of the melt and the crystals, shown in Fig. 10(b,c), the position of the sharp diffraction peaks of the crystal is in general consistence with the diffuse diffraction peak of the liquid, indicating the presence of a structure homology between the melt and the crystal. The relevance of position and hight of FSDP to temperature is presented in Fig. 10(d). The two parameters of FSDP increases linearly with decreasing temperature, which demonstrate that temperature has a certain effect on the local structural unit of the BT2 melt. Interestingly, there is a change in the slope near the critical temperature of supercooled liquid (Δ*T* = Δ*T*_hyp_), which may suggest a pronounced change of viscosity and an increase in the structural order on both short and intermediate length scales^55^.

**Figure 11:**
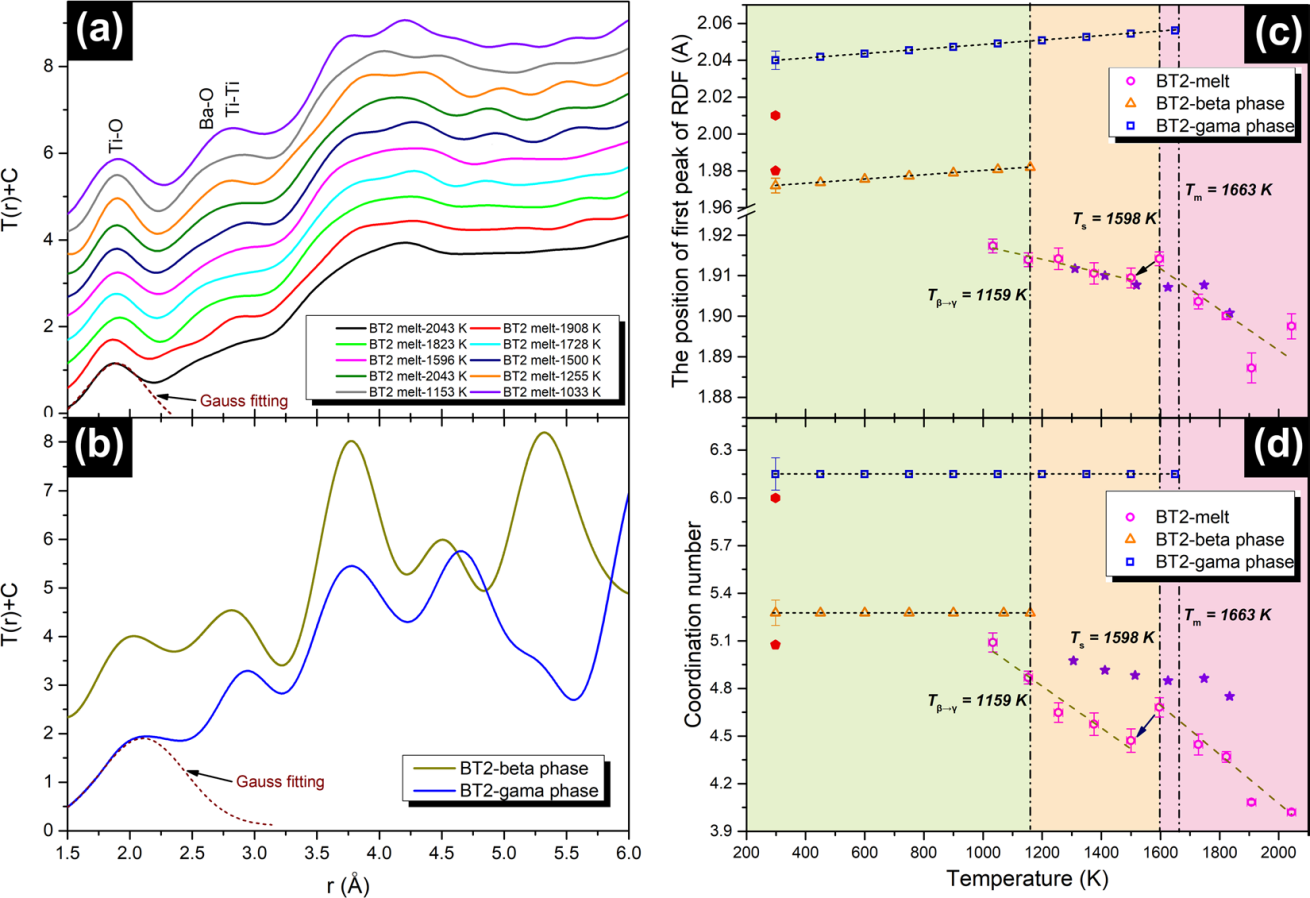
The total correlation function T(r) = 4πrρ_0_g(r): (**a**) BT2 melt (from 2043 K to 1033 K); (**b**) **γ**-BT2 and **β**-BT2 at room temperature. The Ti-O peak in T(r) was fitted by Gaussian function, as shown in (**a**,**b**). The evolution of the Ti-O bond length (**c**) and Ti-O coordination number (**d**) with temperature. The extrapolation of structural data of crystals is based on the thermal expansion^64^. As references, the Ti-O polyhedral bond length and coordination number of melt and crystalline had been reported are also highlighted in (**c**,**d**): purple stars (melt, Alderman^57^), and red hexagon (**γ**-BT2, Yu *et al*.^58^); red pentagon (**β**-BT2, Yu *et al*.^58^).

In order to further investigate the structure homology, the stucture factors are transformed into a total correction function T(r) by Fourier transform. Figure 11(a,b) show the T(r) of the undercooled melt and the crystal, respectively. The first peak observed at ~1.90 Å in T(r) is assigned to the Ti-O pair correlation because the bond length is close to the sum of the ionic radii of oxygen (1.35 Å)^56^ and fivefold titanium (0.54 Å)^56^. In addition, the actual coordination number and bond length of the Ti-O pair can be also obtained from the first peak in T(r) as it does not overlap with other atomic pairs. The bond length of the Ti-O pair is shown in Fig. 11(c). For the BT2 melt, the bond length of Ti-O is in the range of 1.887 Å~1.917 Å in the temperature range between 1033 and 2043 K, which covers the temperature range reported by Alderman (highlighted purple stars)^57^. The Ti-O bond length of BT2 undercooled melt increases with decreasing temperature, which can be described by a linear relationship ${{\rm{r}}}_{{\rm{Ti}}-{\rm{O}}}=1.934-1.666\times {10}^{-5}T\,({\rm{K}})$. The Ti-O bond lengths of **β**-BT2 and **γ**-BT2 (at room temperature) are 1.97 and 2.04 Å, respectively, which are close to those reported by Yu *et al*. (highlighted red hexagon and pentagon)^58^. The coordination number of the Ti-O polyhedra is obtained based on Hennet’s method^55^. After fitting the Ti-O peak with a Gaussian curve *T*_n_(*r*), the coordination number of Ti-O atom pair can be calculated by

9 $$2{\int }_{{r}_{1}}^{{r}_{max}}r{T}_{{\rm{n}}}(r)dr=\frac{{W}_{{\rm{T}}{\rm{i}}-{\rm{O}}}}{{c}_{{\rm{o}}}}{C}_{{\rm{T}}{\rm{i}}}^{{\rm{n}}}(O)$$

where *c*_o_ is the concentration of oxygen. ${W}_{{\rm{Ti}}-{\rm{O}}}$ is the weighting factor for the atom pair Ti-O, which can be calculated by:

10 $${W}_{{\rm{Ti}}-{\rm{O}}}(Q)=2{c}_{{\rm{Ti}}}{c}_{{\rm{O}}}\,{f}_{{\rm{Ti}}}(Q)\,{f}_{{\rm{O}}}(Q)/{({c}_{{\rm{Ba}}}{f}_{{\rm{Ba}}}(Q)+{c}_{{\rm{Ti}}}{f}_{{\rm{Ti}}}(Q)+{c}_{{\rm{O}}}{f}_{{\rm{O}}}(Q))}^{-2}$$

The coordination numbers (CNs) of the Ti-O atom pair for the melt and the crystals of BT2 are shown in Fig. 11(d). The Ti-O coordination number in BT2 melt increases monotonically from 4.02 to 5.09 when the temperature decreases from 2043 to 1033 K despite a drop between 1600 and 1500 K. The Ti-O CNs for **β** and **γ** phase are 5.27 and 6.15, respectively, which are slightly higher than the values measured by powder HEXRD^9^. The linear relationship between Ti-O polyhedra coordination number and the temperature of undercooled BT2 melt can be fitted as ${{\rm{N}}}_{{\rm{Ti}}-{\rm{O}}}=6.393-1.320\times {10}^{-3}T\,({\rm{K}})$. Compared with the results reported by Alderman *et al*.^57^, the values of the Ti-O coordination number in this study are slightly smaller but they change more drastically with temperature, which may be caused by the different cutoffs when calculating the integral (Eq. 9).

**Figure 12:**
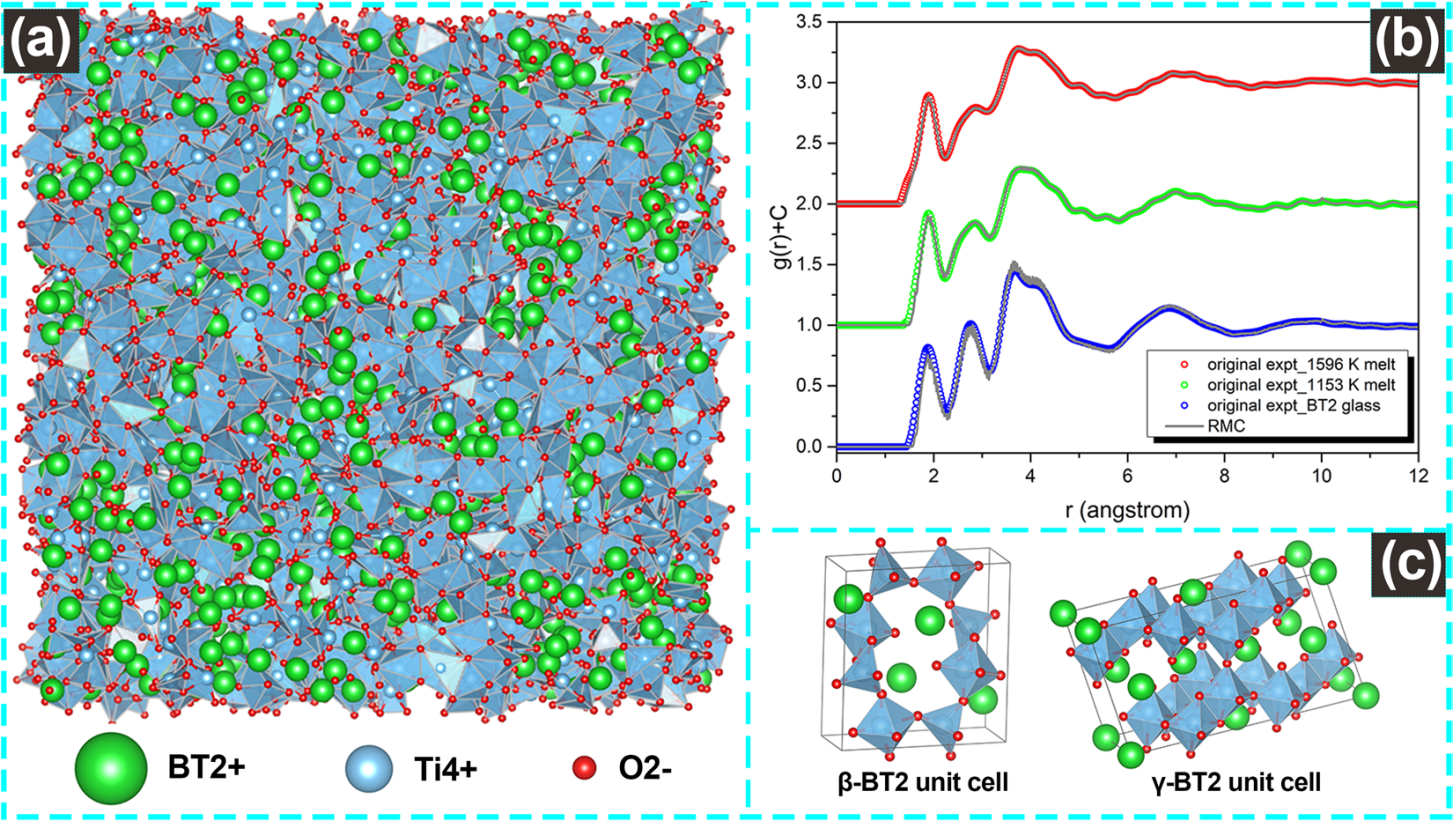
(**a**) The atomic configuration of amorphous state BT2 (glass or melt); (**b**) The fitting between experimental pair distribution function g(r) and the simulated result. (**c**) The unit cell structure of two crystalline BT2 (**β**-BT2 and **γ**-BT2).

Further structural analysis is performed to reveal the atomic confugurations by RMC simulations. The amorphous atomic configurations of two typical undercooling liquid, one is low undercooling liquid and the other one is hypercooling liquid, are constructed based on RMC simulation, a snapshot is shown in Fig. 12(a). Although the atomic configuration of the BT2 melt has never been reported, the structure of the g-BT2 has been extensively studied^9,59^, therefore, we also presented our simulation result of glass for an intuitive comparison. After simulation, the RMC models agree well with the original experimental results, as shown in Fig. 12(b). The atomic configurations of two crystalline BT2 phases were referred from previous work^9,60^, and the crystal unit cell were presented in Fig. 12(c).

**Figure 13:**
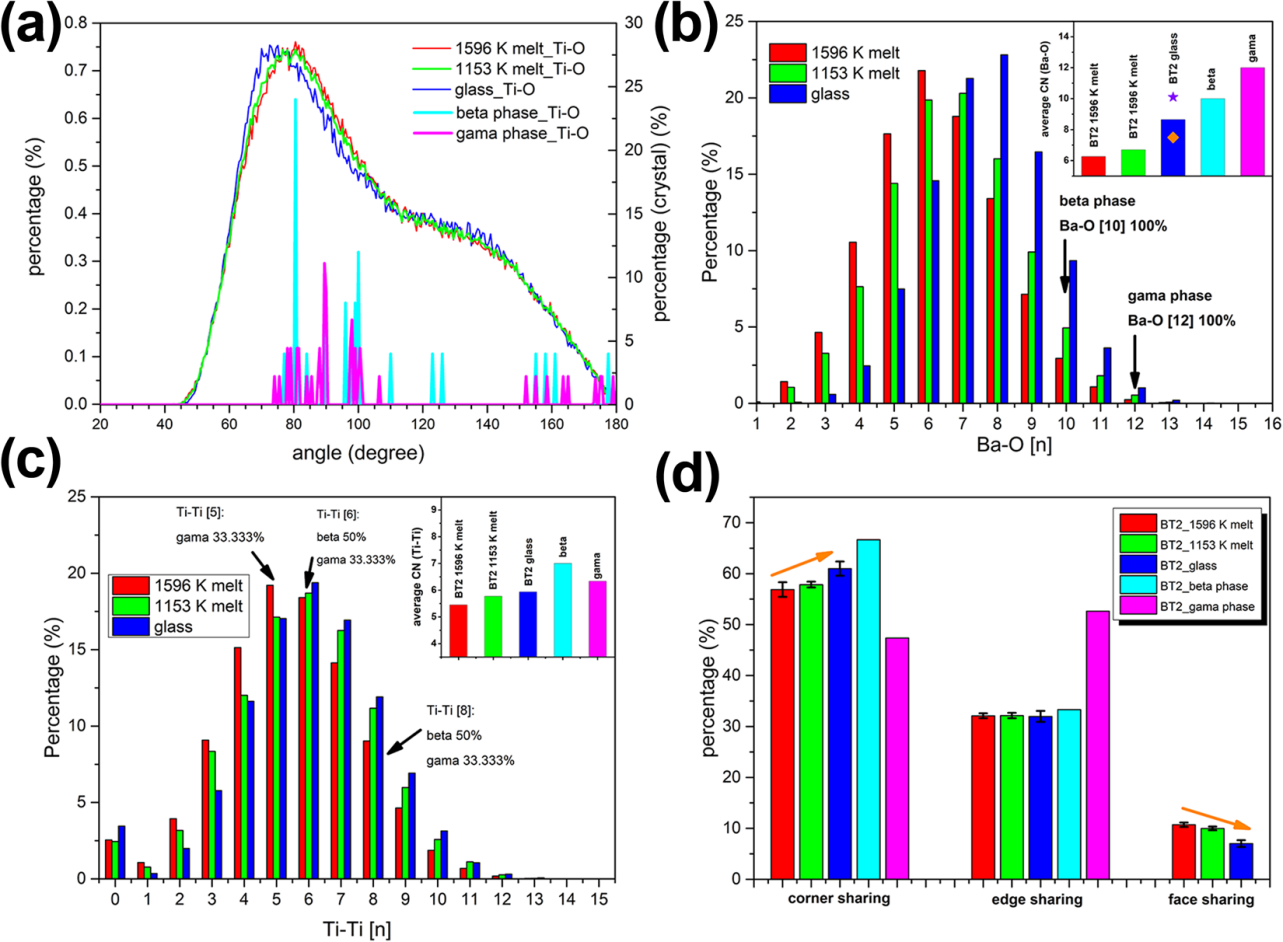
The comparison of structural parameters between amorphous BT2 (melt or glass) and two crystalline phases: (**a**) the bond angle distribution of O-Ti-O pairs; (**b**) the Ba-O polyhedra distribution and the average coordination number (CN) of oxygen around cation Ba (inset); (**c**) the coordination environment between Ti-O polyhedra and the average CN (inset); (**d**) the type of connection modes between Ti-O polyhedra.

Figure 13(a) shows the O-Ti-O bond angle distributions in the first coordination range of Ti. There are two characteristic peaks, at about 80° and 125°, existing in undercooling melt and g-BT2, which agree well with the Ti-O bond angle peaks in **β**-BT2. This result demonstrates that not only the mean bond length and coordination number but also the arrangement of oxygen around Ti, are highly similar between amorphous BT2 state (undercooling liquid or glass) and **β**-BT2 compared with **γ**-BT2 phase. Figure 13(b) gives the distribution of different coordinated Ba-O polyhedra with the first coordination distance of 3.7 Å, and the mean coordination number of oxygens around Ba was obtained by weighted average (inset). The average coordination number of Ba-O is 6.27 in low undercooling melt-BT2 (1596 K, Δ*T* = 67 K), and it increases to 6.71 in hypercooling melt-BT2 (1153 K, Δ*T* = 510 K). The values of Ba-O coordination number in BT2 melt are slightly larger than Alderman’s results calculated by splitting gaussian peaks^57^, which varies from 5.7 to 5.87 in the temperature range of 1300 K to melting point. As for g-BT2, the Ba-O coordination number is 8.65. This value is lower than that obtained by molecular dynamics simulation (N_Ba−O_ = 10.1, marked as a star)^59^ but higher than that reported by Yu *et al*. (N_Ba−O_ = 7.5, marked as a diamond)^9^. Although there is a discrepancy in the Ba-O coordination number calculation, the connectivity of oxygen and barium in undercooling liquid and glass is still closer to **β**-BT2 (CN = 10) than to **γ**-BT2 phase (CN = 12). The stacking of Ti-O polyhedra structural units is analyzed based on the coordination number of Ti around another Ti and the sharing mode between Ti-O polyhedra. Figure 13(c) shows the distribution of Ti-Ti coordination number in undercooling melts, glass and two crystalline phases of BT2. The first coordination range of Ti around another Ti is set as 4.3 Å, the same cutoff distance is used to analyze the amorphous and the crystalline structure. As the temperature decreases (1596 K melt, 1153 K melt and glass), the proportion of highly coordinated Ti-Ti[n] in amorphous BT2 increases, indicating that the structure exhibits a denser stacking. As for the two crystalline BT2 phases, the connectivity between Ti-Ti also shows multiple modes. In **γ**-BT2, the coordination of Ti-Ti consists of 1/3 Ti-Ti[5], 1/3 Ti-Ti[6], and 1/3 Ti-Ti[8]. In contrast, there are 1/2 Ti-Ti[6] and 1/2 Ti-Ti[8] in **β**-BT2. The weighted average coordination numbers of Ti-Ti are shown by the inset figure in Fig. 13(c). In undercooling melt and glass, the Ti-Ti coordination number are 5.45 (1596 K melt), 5.77 (1153 K melt) and 5.94 (glass) respectively. A denser stacking of Ti-O polyhedra is found in **β**-BT2 than in **γ**-BT2 due to a larger average coordination number of Ti-Ti. The proportion of three types of sharing modes between Ti-O polyhedra (corner, edge and face sharing) are presented in Fig. 13(d). Corner and edge sharing are the prominent sharing modes in both of BT2 amorphous phases and crystalline phases, however, the face sharing only exists in amorphous BT2 phases. Comparing from the proportion of sharing modes, it’s clear that the sharing modes in BT2 undercooling melt and glass are more like **β**-BT2 phase rather than **γ**-BT2, which indicates that less bridging of Ti-O polyhedra needs to be broken when **β**-BT2 nucleates from undercooling liquid or glass.

From the above comparison of the structural parameters between BT2 melt, glass and two crystalline phases, it can be concluded that the structural differences between undercooling liquid (or glass) and crystalline phases have always existed, but not to the same extent. This structural dissimilarity depicts the barrier for nucleation at the atomic level, therefore, different nucleation tendency of two crystalline phases can be predicted. In view of the arrangement of oxygens around Ti, the connectivity of oxygen and Ba, and the stacking of Ti-O polyhedra, the structural dissimilarity between **β**-BT2 phase and BT2 undercooling liquid (and glass) is smaller than the **γ**-BT2. These structural information covers the minimum size of crystal nucleus (unit cell) in the spatial scale, which positively indicates that **β** phase nuclei are more likely to be incubated from the undercooling melt.

It is clear that there exists a strong relationship between the structural similarity and the nucleation scenario in BT2 crystallization process. However, how does the analogous structure contribute to the preferential formation of **β**-BT2 crystal nuclei? Determination the nucleation barrier is one of the major issues in the theoretical study of nucleation. In the nucleation of quasicrystal, Kelton *et al*.^50,61^ proposed that the structural homology between icosahedra in undercooling melt and quasicrystal decreases the barrier for the nucleation of the metastable quasicrystal. However, complex local ordered structure and component differences between undercooling melt and quasicrystal hinder further elaboration of this process. In this study, the BT2 crystalline phases and undercooling melt of the same component can avoid the influence of component diffusion and segregation on nucleation, which facilitates to establish a relationship between the structural homology and the nucleation barrier.

Although the nucleation of BT2 presents a non-classical two step pathway, the precipitation of pre-nucleation clusters^18^ or the preferential nucleation of metastable intermediate phase^20^ are believed to obey the classical nucleation theory (CNT). Therefore, the critical nucleation energy is describled as:

11 $${\rm{\Delta }}{G}^{\ast }=\frac{16}{3}\frac{\pi {\gamma }_{{\rm{sl}}}^{3}}{{\rm{\Delta }}{G}_{{\rm{v}}}^{2}}=\frac{16}{3}\frac{\pi {\gamma }_{{\rm{sl}}}^{3}{T}_{{\rm{f}}}^{2}}{{\rm{\Delta }}{H}_{f}^{2}{\rm{\Delta }}{T}^{2}}$$

The Skapski’s next-neighbors theory^62^ is used for calculating the interfacial energy due to structural parameters are contained in this model.

12 $${\gamma }_{sl}=\frac{{Z}_{{\rm{s}}}-{Z}_{{\rm{l}}}}{{Z}_{{\rm{s}}}}\frac{{\rm{\Delta }}{H}_{{\rm{f}}}}{{N}^{1/3}{V}_{{\rm{m}}}^{2/3}}+\frac{2}{3}({(\frac{{\rho }_{{\rm{s}}}}{{p}_{{\rm{l}}}})}^{2/3}-1){\sigma }_{{\rm{l}}}+\frac{{T}_{{\rm{f}}}}{{A}_{{\rm{s}}}}({\rm{\Delta }}{S}_{{\rm{l}}}-{\rm{\Delta }}{S}_{{\rm{s}}})$$

Where *Z*_S_ is the number of the nearest neighbors in the interior of the crystal, and *Z*_1_ are the number of nearest neighbor in the surface of nucleated crystal. *N* is Avogadro constant, *V*_m_ is the molar volume of nucleated crystals, *ρ*_s_ and *ρ*_1_ are the density of crystal and liquid, *σ*_1_ is the surface tension of liquid. Δ*S*_s_ and Δ*S*_1_ are the entropy surpluses at the surface of crystal and liquid. *A*_s_ is the molar surface of solid. The expression (12) can be simplified through bypassing the third term, which is much smaller comparing with the sum of two others^62^.

As for ionic liquid like oxide-ceramic melt, strongly bonded metal cation-oxygen polyhedra are prominent SRO clusters, which are treated as local structural unit. In BT2 melt, the Ti-O polyhedra instead of the Ba-O polyhedra is assigned as structural unit due to much a stronger chemical bond of the Ti-O pair than the Ba-O pair. We assumed that the structural unit Ti-O polyhedra mainly contribute to the nucleation interfacial energy because Ti-O polyhedra are the fundamental bricking motifs in both the melt and the crystalline phase. Although the connectivity of oxygens and Ba, as well as the sharing modes between Ti-O polyhedral, are also related to the nucleation interfacial energy, here we only consider the structural parameters of Ti-O polyhedra into Eq. (12) for the interface energy calculation based on the simplified assumption. *Z*_s_ and *Z*_1_ are replaced by the coordination number of Ti-O ployhedra in **γ**, **β**-BT2 and melt, respectively. The experimental value of the surface tension *σ*_1_ of the undercooled BT melt is used in the calculation^63^, which avoids a subversive conclusion. All the parameters used in critical nucleation energy calculation are list in Table 2. According to CNT, a lower nucleation energy can promote corresponding crystal nuclei preferential formation. Thus, the ratio of the critical nucleation energy between the **γ** and **β** phases was calculated, which qualitatively reflects the nucleation sequence.

13 $${(\frac{{\rm{\Delta }}{G}_{{\rm{\gamma }}}^{\ast }}{{\rm{\Delta }}{G}_{{\rm{\beta }}}^{\ast }})}^{1/3}=\frac{{\gamma }_{{\rm{sl}}}^{{\rm{\gamma }}}}{{\gamma }_{{\rm{sl}}}^{{\rm{\beta }}}}{(\frac{{\rm{\Delta }}{H}_{{\rm{f}}}^{{\rm{\beta }}}}{{\rm{\Delta }}{H}_{{\rm{f}}}^{{\rm{\gamma }}}})}^{2/3}=\frac{\frac{{Z}_{{\rm{\gamma }}}-{Z}_{{\rm{l}}}}{{Z}_{{\rm{\gamma }}}}\frac{{\rm{\Delta }}{H}_{{\rm{f}}}^{{\rm{\gamma }}}{\rho }_{{\rm{\gamma }}}^{2/3}}{{N}^{1/3}{M}^{2/3}}+\frac{2}{3}({(\frac{{\rho }_{{\rm{\gamma }}}}{{p}_{{\rm{l}}}})}^{2/3}-1){\sigma }_{{\rm{l}}}}{\frac{{Z}_{{\rm{\beta }}}-{Z}_{{\rm{l}}}}{{Z}_{{\rm{\beta }}}}\frac{{\rm{\Delta }}{H}_{{\rm{f}}}^{{\rm{\beta }}}{\rho }_{{\rm{\beta }}}^{2/3}}{{N}^{1/3}{M}^{2/3}}+\frac{2}{3}({(\frac{{\rho }_{{\rm{\beta }}}}{{p}_{{\rm{l}}}})}^{2/3}-1){\sigma }_{{\rm{l}}}}{(\frac{{\rm{\Delta }}{H}_{{\rm{f}}}^{{\rm{\beta }}}}{{\rm{\Delta }}{H}_{{\rm{f}}}^{{\rm{\gamma }}}})}^{2/3}$$

**Table 2:** The value of parameters used in the calculation the ratio of critical nucleation energy of γ and β-BT2.

Parameter	Symbol [unit]	Value
Ti-O coordination number of crystal	*Z*_γ_, *Z*_β_	6.15, 5.27
Ti-O coordination number of undercooled melt	*Z*_1_	5.496 − 4.361 × 10^−4^T
Enthalpy of fusion	${\rm{\Delta }}{H}_{{\rm{f}}}^{{\rm{\gamma }}}$, ${\rm{\Delta }}{H}_{{\rm{f}}}^{{\rm{\beta }}}$ [kJ·mol^−1^]	81.4, 71.21
Density (crystal)	*ρ*_γ_, *ρ*_β_ [kg·m^−3^]	5.12, 4.73^57^
Density (melt)	*ρ*_1_ [kg·m^−3^]	4.82-0.0004T^57^
Molar mass	M [g/mol]	313.13
Surface tension of melt	σ_1_ [mN·m^−1^]	349-0.03(T − T_f_)^63^

**Figure 14:**
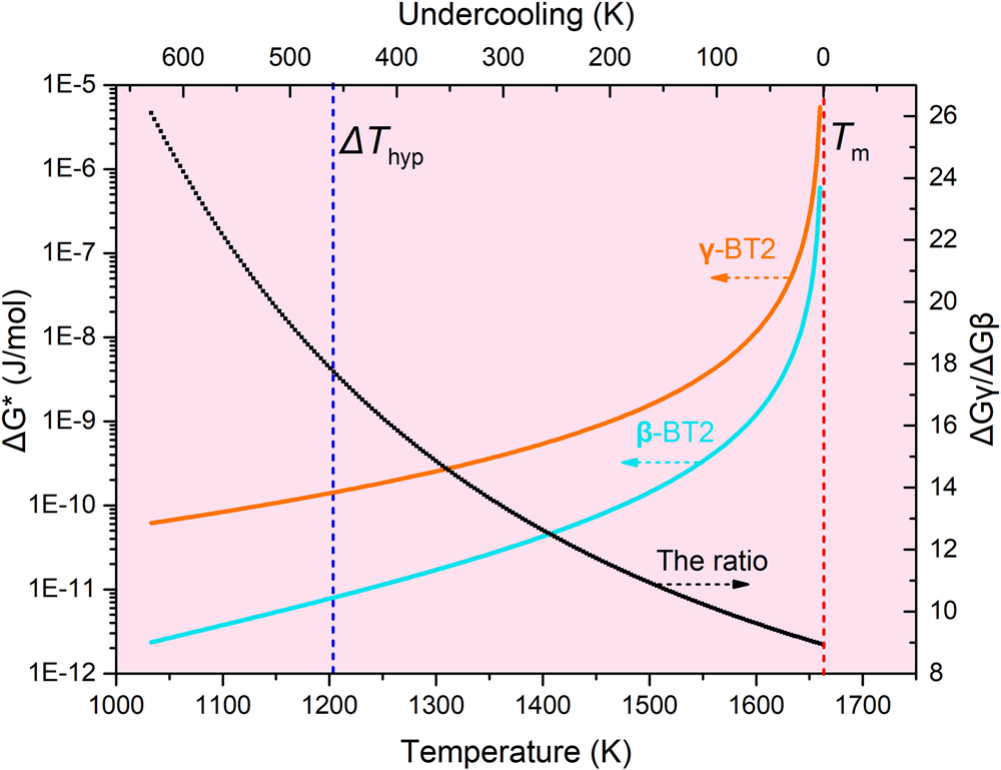
The critical nucleation energy of **γ**- and **β**-BT2 in the temperature range of 1033 K to 1663 K (0.62*T*_m_ − *T*_m_), which was calculated according to CNT (Eq. (11)), with the expression of the interfacial energy (Eq. (12)). Within this temperature range, the ratio of critical nucleation energy of both phases was present in black line.

The calculation result of the critical nucleation energy and the ratio are shown in Fig. 14. Within the temperature range of 0.62 *T*_m_ to *T*_m_, the critical nucleation energy of **β**-BT2 is less than **γ**-BT2, which means **β** phase will preferentially nucleate prior to **γ** phase due to a relatively lower nucleation barrier. Furthermore, the ratio $\frac{{\rm{\Delta }}{G}_{{\rm{\gamma }}}^{\ast }}{{\rm{\Delta }}{G}_{{\rm{\beta }}}^{\ast }}$ is larger than 8 and increases with increasing undercooling, which indicates a rising chance of **β**-BT2 nucleation preferentially in deep undercooled melt.

From the calculation and comparison of the critical nucleation energy, it is found that despite a lower nucleaion driving force of **β**-BT2 than **γ**-BT2 $({\rm{\Delta }}{H}_{{\rm{f}}}^{{\rm{\beta }}} < {\rm{\Delta }}{H}_{{\rm{f}}}^{{\rm{\gamma }}})$, the much lower nucleation interfacial energy contributes to reducing the nucleation barrier, and consequently  leading to the preferential nucleation of **β**-BT2. Therefore, the strongly kinetic stability of this metastable intermediate is attributed to a lower interfacial energy, which stems from the structural similarity between its crystal and undercooling melt.

## Conclusion

The nucleation pathway of a novel ferroelectric BaTi_2_O_5_ during the crystallization process from cooling melt is investigated in this work. ADL containerless processing is employed to provide a large controllable undercooling range and the crystallization is achieved by triggering at various undercoolings.  Fundamental thermodynamic data of BT2, are estimated and the critical undercoolng ∆T_hyp_ of hypercooling is calculated as 459.8 K, which agrees with the triggering experiment. A polymorphic transition is observed: the ferroelectric **γ**-BT2 can be obtained at below ∆T_hyp_, and the metastable polymorphs (**β**-BT2) appears under hypercooling condition. Furthermore, **β** phase gradually replaces **γ** phase as the main phase when the undercooling increases. *In situ* real-time HEXRD reveals a non-classical stepping nucleation pathway of BT2 during undercooling solidification. The metastable **β**-BT2 phase preferentially nucleates from the undercooled melt, and the ferroelectric **γ**-BT2 grows from **β**-BT2 by solid-state phase transition. This stepping nucleation provides a interesting hybride microstructure, where **β** phase and **γ** phase presents discrete regional distribution.

A structural similarity of SRO structural unit (Ti-O polyhedra) between the undercooled liquid and the metastable **β** phase is observed based on diffraction data. The average bond length r_Ti−O_ and coordination number N_Ti−O_ of Ti-O polyhedra in undercooled melt (1033 K~1663 K) were measured at r_Ti−O_ = 1.934 − 1.666 × 10^−5^*T* (K, Å) and ${{\rm{N}}}_{{\rm{Ti}}-{\rm{O}}}=6.393-1.320\times {10}^{-3}T\,({\rm{K}})$ respectively, which are closer to the structural parameters of **β** phase (${{\rm{r}}}_{{\rm{Ti}}-{\rm{O}}}=1.972\,{\rm{\AA }}$, ${{\rm{N}}}_{{\rm{Ti}}-{\rm{O}}}=5.277$) than to those of **γ** phase (${{\rm{r}}}_{{\rm{Ti}}-{\rm{O}}}=2.04\,{\rm{\AA }}$, ${{\rm{N}}}_{{\rm{Ti}}-{\rm{O}}}=6.15$). Moreover, the structural homology between the undercooled liquid and the crystalline phases are further confirmed by RMC simulations and atomic configurations of **β**, **γ**-BT2 unit cell. In view of the arrangement of oxygens around Ti, the connectivity of oxygen and Ba, and the stacking of Ti-O polyhedra, the structure of the BT2 undercooled melt is more like the **β**-BT2 rather than **γ**-BT2 phase. This structural similarity coupled with CNT is used to explain the preferential nucleation of metastable **β**-BT2. The results demonstrate that similar structural unit reduces the nucleation interfacial energy and therefore contributes to a strong kinetic stability of **β** phase embryos.

## Data Availability

The datasets generated during the current study are available from the corresponding author on reasonable request.
